# Supplementary material for: Effects of different nutrition interventions on sarcopenia criteria in older people: A study protocol for a systematic review of systematic reviews with meta-analysis
Source: PLoS One. 2024 May 10;19(5):e0302843. doi: 10.1371/journal.pone.0302843 (PMC11086819; doi:10.1371/journal.pone.0302843)
Supplement: S1 Appendix — (DOCX) [file pone.0302843.s001.docx]

# Appendix 1. Preferred reporting items for systematic review and meta-analysis protocols (PRISMA-P) pages for this study.

| Section and topic | Item No | Page |
| --- | --- | --- |
| ADMINISTRATIVE INFORMATION | | |
| Title: |  |  |
| Identification | 1a | 1 |
| Update | 1b | Not applicable |
| Registration | 2 | 4 |
| Authors: |  |  |
| Contact | 3a | 1 |
| Contributions | 3b | 1 |
| Amendments | 4 | Not applicable |
| Support: |  |  |
| Sources | 5a | 10 |
| Sponsor | 5b | Not applicable |
| Role of sponsor or funder | 5c | 10 |
| INTRODUCTION | | |
| Rationale | 6 | 3 |
| Objectives | 7 | 3 |
| METHODS | | |
| Eligibility criteria | 8 | 4-5 |
| Information sources | 9 | 4 and 6 |
| Search strategy | 10 | Appendix 2 |
| Study records: |  |  |
| Data management | 11a | 5 and 7 |
| Selection process | 11b | 5 |
| Data collection process | 11c | 7-9 |
| Data items | 12 | 7-9 |
| Outcomes and prioritization | 13 | 7-9 |
| Risk of bias in individual studies | 14 | 6 |
| Data synthesis | 15a | 7 |
|  | 15b | 7-9 |
|  | 15c | 9 |
|  | 15d | Not applicable |
| Meta-bias(es) | 16 | 6 |
| Confidence in cumulative evidence | 17 | Not applicable |
