# Supplementary material for: Effects of different nutrition interventions on sarcopenia criteria in older people: A study protocol for a systematic review of systematic reviews with meta-analysis
Source: PLoS One. 2024 May 10;19(5):e0302843. doi: 10.1371/journal.pone.0302843 (PMC11086819; doi:10.1371/journal.pone.0302843)
Supplement: S2 Appendix — (DOCX) [file pone.0302843.s002.docx]

# Appendix 2. Key words, MeSH, subheadings and search strategy for the systematic review of systematic reviews.

| MeSH | Subheadings |
| --- | --- |
| Nutrition Therapy | Diet Therapy  Caloric Restriction  Diet, Carbohydrate Loading  Diet, Carbohydrate-Restricted  Diet, High-Protein + |
| Diet, Food, and Nutrition | Dietary Carbohydrates  Dietary Fats  Dietary Fiber  Dietary Proteins  Dietary Supplements  [Nutrients](https://www.ncbi.nlm.nih.gov/mesh/2028222)  Diet  [Eating](https://www.ncbi.nlm.nih.gov/mesh/68004435)  Nutritional Requirements  Nutritional Status |
| Nutritional Sciences | Dietetics |
| Sports Nutritional Sciences |  |
| Diet, Healthy |  |
| Diet |  |
| Proteins | Dietary Proteins  Animal Proteins, Dietary  Fruit Proteins  Grain Proteins  Plant Proteins, Dietary  Muscle Proteins |
| Dietary Supplements | Supplementation |
| Nutritional Requirements | Recommended Dietary Allowances |
| dietary patterns |  |
| Food |  |
| Geriatric nutrition |  |
| Nutritional Status |  |
| nutrition intervention |  |
| diet quality |  |

Basic strategy:

("sarcopenia"[MeSH Terms] OR "sarcopenia"[All Fields]) AND ("aged"[MeSH Terms] OR "aged"[All Fields] OR ("aging"[MeSH Terms] OR "aging"[All Fields] OR "ageing"[All Fields]) OR ("aged"[MeSH Terms] OR "aged"[All Fields] OR "elderly"[All Fields] OR "elderlies"[All Fields] OR "elderly s"[All Fields] OR "elderlys"[All Fields]) OR ("older"[All Fields] OR "olders"[All Fields]))

Pubmed/MedLine strategy:

Search (((((((((sarcopenia[MeSH Terms]) OR sarcopenia)) AND ((((((((((((olders) OR aging) OR older) OR aged) OR aging[MeSH Terms]) OR ageing) OR aged[MeSH Terms]) OR ageing[MeSH Terms]) OR elderly) OR elderlies) OR elderly[MeSH Terms]) OR elderlys)) AND ((((((((((((((((((((((((((((((((((((Nutrition Therapy)[Title] OR Diet, High-Protein +)[Title] OR Diet Therapy)[Title] OR Caloric Restriction)[Title] OR Diet, Carbohydrate Loading)[Title] OR Diet, Carbohydrate-Restricted)[Title] OR (Diet, Food,[Title] AND Nutrition))[Title] OR Dietary Carbohydrates)[Title] OR Dietary Fats)[Title] OR Dietary Fiber)[Title] OR Dietary Proteins)[Title] OR Dietary Supplements)[Title] OR Nutrients)[Title] OR Diet)[Title] OR Eating)[Title] OR Nutritional Requirements)[Title] OR Nutritional Status)[Title] OR Nutritional Sciences)[Title] OR Dietetics)[Title] OR Sports Nutritional Sciences)[Title] OR Diet, Healthy)[Title] OR Proteins)[Title] OR Dietary Proteins)[Title] OR Animal Proteins, Dietary)[Title] OR Fruit Proteins)[Title] OR Grain Proteins)[Title] OR Plant Proteins, Dietary)[Title] OR Muscle Proteins)[Title] OR Dietary Supplements)[Title] OR Supplementation)[Title] OR Nutritional Requirements)[Title] OR Recommended Dietary Allowances)[Title] OR dietary patterns)[Title] OR Food)[Title] OR Geriatric nutrition)[Title] OR Nutritional Status[Title])) AND (((Systematic Review [Publication Type]) OR Systematic Review) OR Review, Systematic)) AND ((sarcopenia[MeSH Terms]) OR sarcopenia)) AND ((((((((((((olders) OR aging) OR older) OR aged) OR aging[MeSH Terms]) OR ageing) OR aged[MeSH Terms]) OR ageing[MeSH Terms]) OR elderly) OR elderlies) OR elderly[MeSH Terms]) OR elderlys)) AND ((((((((((((((((((((((((((((((((((((Nutrition Therapy)[Abstract] OR Diet, High-Protein +)[Abstract] OR Diet Therapy)[Abstract] OR Caloric Restriction)[Abstract] OR Diet, Carbohydrate Loading)[Abstract] OR Diet, Carbohydrate-Restricted)[Abstract] OR (Diet, Food,[Abstract] AND Nutrition))[Abstract] OR Dietary Carbohydrates)[Abstract] OR Dietary Fats)[Abstract] OR Dietary Fiber)[Abstract] OR Dietary Proteins)[Abstract] OR Dietary Supplements)[Abstract] OR Nutrients)[Abstract] OR Diet)[Abstract] OR Eating)[Abstract] OR Nutritional Requirements)[Abstract] OR Nutritional Status)[Abstract] OR Nutritional Sciences)[Abstract] OR Dietetics)[Abstract] OR Sports Nutritional Sciences)[Abstract] OR Diet, Healthy)[Abstract] OR Proteins)[Abstract] OR Dietary Proteins)[Abstract] OR Animal Proteins, Dietary)[Abstract] OR Fruit Proteins)[Abstract] OR Grain Proteins)[Abstract] OR Plant Proteins, Dietary)[Abstract] OR Muscle Proteins)[Abstract] OR Dietary Supplements)[Abstract] OR Supplementation)[Abstract] OR Nutritional Requirements)[Abstract] OR Recommended Dietary Allowances)[Abstract] OR dietary patterns)[Abstract] OR Food)[Abstract] OR Geriatric nutrition)[Abstract] OR Nutritional Status[Abstract])) AND (((Systematic Review [Publication Type]) OR Systematic Review) OR Review, Systematic)

Embase strategy:

('sarcopenia'/exp OR 'sarcopenia') AND ('aged'/exp OR aged) AND ('nutrition'/exp OR nutrition) AND ([cochrane review]/lim OR [systematic review]/lim OR [meta analysis]/lim)

Scopus strategy:

[( TITLE-ABS-KEY ( nutrition  AND therapy )  AND  TITLE-ABS-KEY ( aged )  OR  TITLE-ABS-KEY ( elderly )  AND  TITLE-ABS-KEY ( sarcolemma )  AND  TITLE-ABS-KEY ( systematic  AND review ) )](https://www-scopus.ez41.periodicos.capes.gov.br/results/documentSpellSuggest.uri?sort=plf-f&src=s&st1=Nutrition+Therapy&st2=aged&searchTerms=elderly%3f%21%22*%24sarcopenia%3f%21%22*%24systematic+review%3f%21%22*%24&sid=a9c5ca4f6c670552ae8ff589d35a7500&sot=b&sdt=b&sl=168&s=%28+TITLE-ABS-KEY+%28+Nutrition+Therapy+%29+AND+TITLE-ABS-KEY+%28+aged+%29+OR+TITLE-ABS-KEY+%28+elderly+%29+AND+TITLE-ABS-KEY+%28+sarcolemma+%29+AND+TITLE-ABS-KEY+%28+systematic+review+%29+%29&origin=resultslist)

Cinahl strategy:

sarcopenia or sarcopenic or muscle weakness or muscular atrophy or muscle loss and aged or elderly or senior or older people or geriatric and nutrition or diet or food or nourishment or food intake or eating

Web of Science strategy:

((ALL=(sarcopenia)) AND ALL=(nutrition)) AND ALL=(aged)

Cochrane strategy:

sarcopenia in Title Abstract Keyword AND "aging" in Title Abstract Keyword OR aged in Title Abstract Keyword AND "nutrition" in Title Abstract Keyword - (Word variations have been searched)
